# Computational design and characterization of nanobody-derived peptides that stabilize the active conformation of the β_2_-adrenergic receptor (β_2_-AR)

**DOI:** 10.1038/s41598-019-52934-8

**Published:** 2019-11-12

**Authors:** Milan Sencanski, Sanja Glisic, Marko Šnajder, Nevena Veljkovic, Nataša Poklar Ulrih, Janez Mavri, Milka Vrecl

**Affiliations:** 1 Center for Multidisciplinary Research, Institute of Nuclear Sciences VINCA, University of Belgrade, Belgrade, Serbia; 2 Biotechnical Faculty, University of Ljubljana, Ljubljana, Slovenia; 3 Laboratory of Computational Biochemistry and Drug Design, National Institute of Chemistry, Ljubljana, Slovenia; 4 Institute of Preclinical Sciences, Veterinary Faculty, University of Ljubljana, Ljubljana, Slovenia

**Keywords:** Bioinformatics, Bioinformatics, Protein design, Protein design, Molecular modelling

## Abstract

This study aimed to design and functionally characterize peptide mimetics of the nanobody (Nb) related to the β_2_-adrenergic receptor (β_2_-AR) (nanobody-derived peptide, NDP). We postulated that the computationally derived and optimized complementarity-determining region 3 (CDR3) of Nb is sufficient for its interaction with receptor. Sequence-related Nb-families preferring the agonist-bound active conformation of β_2_-AR were analysed using the informational spectrum method (ISM) and β_2_-AR:NDP complexes studied using protein-peptide docking and molecular dynamics (MD) simulations in conjunction with metadynamics calculations of free energy binding. The selected NDP of Nb71, designated P3, was 17 amino acids long and included CDR3. Metadynamics calculations yielded a binding free energy for the β_2_-AR:P3 complex of ΔG = (−7.23 ± 0.04) kcal/mol, or a Kd of (7.9 ± 0.5) μM, for T = 310 K. *In vitro* circular dichroism (CD) spectropolarimetry and microscale thermophoresis (MST) data provided additional evidence for P3 interaction with agonist-activated β_2_-AR, which displayed ~10-fold higher affinity for P3 than the unstimulated receptor (MST-derived EC_50_ of 3.57 µM *vs*. 58.22 µM), while its ability to inhibit the agonist-induced interaction of β_2_-AR with β-arrestin 2 was less evident. In summary, theoretical and experimental evidence indicated that P3 preferentially binds agonist-activated β_2_-AR.

## Introduction

Nanobodies (Nbs) are recombinant, antigen-specific, single-domain, variable fragments of camelid heavy-chain-only antibodies with a broad range of diagnostic, therapeutic and research applications^1^, including studies of G-protein-coupled receptors (GPCRs). GPCRs comprise the largest family of cell-surface receptors, as well as the most intensively studied drug target family, with implications in almost every major disease category^2^. In particular, GPCR crystallography has experienced impressive progress in recent years. Since the structures of rhodopsin and β_2_-adrenergic receptor (β_2_-AR) were resolved in 2000^3^ and 2007^4,5^, respectively, over 200 structures of more than 50 GPCRs have been solved (reviewed in^6^); yet data about the conformational changes associated with their activation is still sparse. As the biological activity induced by the binding of a ligand to orthosteric or allosteric sites on a GPCR is mediated by the stabilization of specific receptor conformations^7^, the emerging theme in GPCR activation/signalling is the role of the structural conformation of the receptor in G-protein/effector protein selection. The approaches used to obtain detailed descriptions of GPCR activation dynamics include molecular simulations and the use of Nbs that bind conformational epitopes, which occur only in native proteins^8,9^. Nbs were instrumental in solving (i) the first structure of activated β_2_-AR in complex with the Nb designated Nb80 and slowly dissociating agonist BI167107^10^, (ii) agonist-occupied β_2_-AR:Gs heterotrimer complex^10^ and (iii) β_2_-AR in complex with its low-affinity agonist adrenaline and the nanobody Nb6B9, which exhibited improved affinity and slower dissociation^11^. The same approach also facilitated the crystallization of agonist-bound active-state structures of other GPCRs (for reviews see^8,12,13^). Additionally, β_2_-AR-specific Nbs transiently expressed as “intrabodies” in HEK-293 cells retain their conformational specificity and have been used as a tool to study GPCR signalling via G-protein and β-arrestin recruitment^14^. Nbs that stabilize the active conformation, such as Nb80, bind to the intracellular domain of a GPCR that is otherwise occupied by Gα subunit or β-arrestin^15^, primarily through the third complementarity-determining region (CDR3), whereas CDR1 should stabilize only this interaction^16^. Therefore, we hypothesized that approximately only 25% of the length of original Nbs that is computationally derived and optimized is sufficient for its interaction with the receptor. The main objective of our study was, therefore, the computational design and functional characterization of peptide mimetics of the Nb CDR3 related to β_2_-AR, *i.e*., Nb-derived peptides (NDPs). We used the following computational approaches to test our hypothesis: (i) the informational spectrum method (ISM), a virtual spectroscopy method for investigations of protein-protein interactions and the structure/function relationship of proteins^17,18^ to design NDPs and to arrange a sequence of amino acids of NDPs that are functionally related to Nbs from the camelid family that are related to β_2_-AR, with reference to the informational properties of Nbs; (ii) explicit membrane molecular dynamics (MD) of derived NDPs docked into the intracellular space of the β_2_-AR active conformation to compute the protein-peptide binding free energy; and (iii) a novel computational approach in molecular dynamics stimulation that was introduced in 2002, known as metadynamics^19,20^ and implemented in the NAMD program^21^ with a CHARMM27 force field^22,23^, to examine the whole molecular conformational space and calculate the free energy during MD simulation. One selected computationally characterized NDP was then experimentally tested (i) by assessing its ability to bind β_2_-AR using techniques for studying protein-protein interactions, *i.e*., circular dichroism (CD) spectroscopy to detect changes in the conformation of interacting proteins^24^ and microscale thermophoresis (MST), a powerful analytical technique for characterizing biomolecular interactions based on the movement of molecules in microscopic temperature gradients (reviewed in^25^); and (ii) by interfering with its function using the previously developed bioluminescence resonance energy transfer (BRET)-based β-arrestin 2 recruitment assay^26^. Based on our results, molecular simulation-based theoretical calculations of binding free energy values are highly consistent with the experimental data, particularly the MST-derived half-maximal effective concentration (EC_50_) of the derived P3 for the agonist-activated β_2_-AR.

## Results and Discussion

### ISM analysis of Nbs and the interaction between β_2_-AR and Nbs

**Figure 1:**
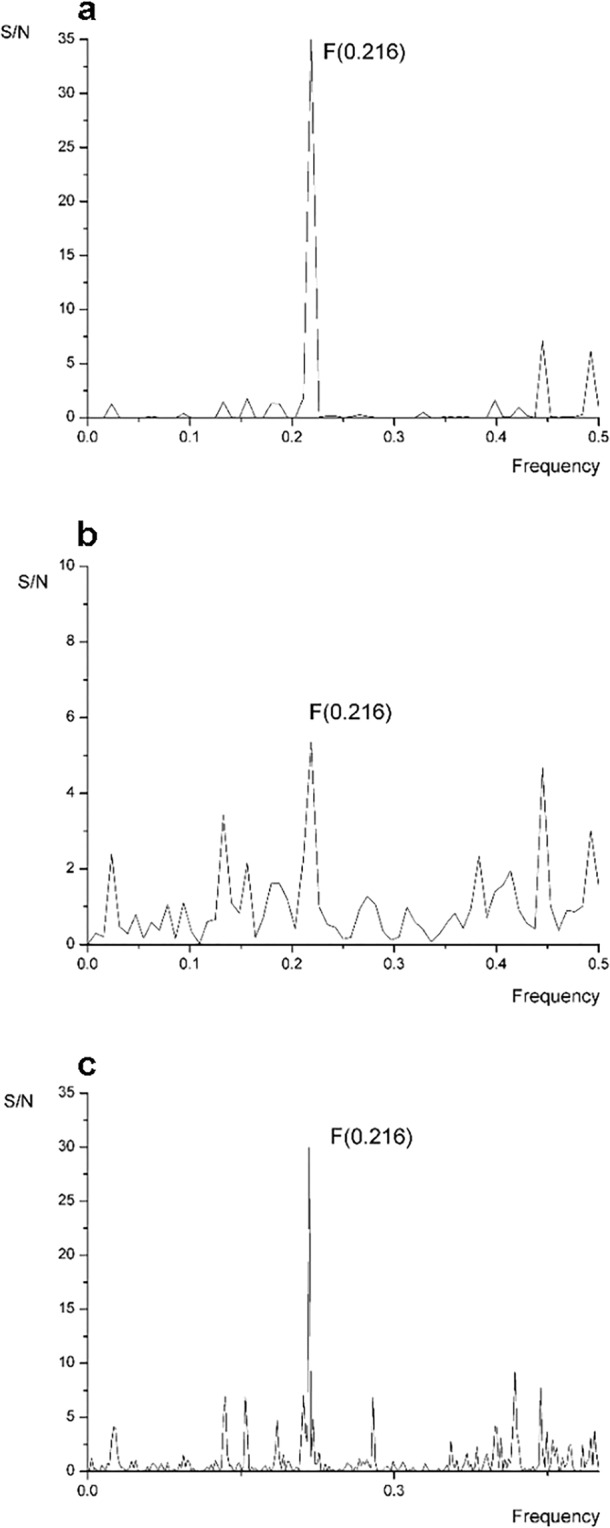
Bioinformatics analysis of Nbs and β_2_-AR and Nb71 using ISM. (**a**) Cross-spectral analysis (CIS) of Nb80, Nb84 and Nb71 stabilizing the active conformation of β_2_-AR, with the characteristic peak at F(0.216). (**b**) IS of Nb71 and (**c**) CS of β2-AR and Nb71. The CS of β2-AR and Nb71 showed a common peak corresponding to the IS frequency F(0.216).

ISM was employed to identify common informational characteristics of Nbs in terms of their preference for agonist-occupied β_2_-AR and information about the characteristics of the interaction between β_2_-AR and the Nbs, as well as to identify the key domain of Nb involved in receptor targeting (Fig. 1). Based on CDR3 conservation, β_2_-AR Nbs were classified into four distinct families [A, B, C, and miscellaneous (MISC)]. Nbs that showed a clear preference for agonist (BI-167107)-occupied β_2_-AR belonged to family B^14^. Here, we analysed the extensively characterized Nb80 from family B that binds agonist-activated β_2_-AR^10^ and Nb84 and Nb71 from Nb families C and MISC, respectively, the latter two of which display a preference for binding active β_2_-AR conformations^14^. Common biological characteristics of the group of proteins that share common information are represented by peaks in their consensus informational spectrum (CIS)^18^. A cross-spectral analysis of Nbs stabilizing an active β_2_-AR conformation showed that Nbs shared common information corresponding to the informational spectrum (IS) frequency F(0.216) (Fig. 1a). An ISM analysis of individual spectra of Nbs that bound the β_2_-AR only in the presence of agonist, Nb80 and Nb71 (Fig. 1b), revealed the same dominant IS peak at the frequency F(0.216) in both individual spectra. Furthermore, we performed a cross-spectral analysis of β_2_-AR and Nb71 and identified that these two molecules shared the same common information corresponding to the IS frequency F(0.216), indicating their interaction (Fig. 1c). This finding prompted us to speculate that the common information for Nbs is also the most important for the interaction of Nb71 with the β2-AR. In a subsequent analysis, we wanted to determine the Nb domains that were most important for the identified information feature because they would be a key part of the Nb in its interaction with the receptor. We assumed that this isolated portion of the Nb would be sufficient for the interaction with the β_2_-AR and for stabilizing an active receptor conformation.

### Identification of the key protein domain responsible for the interaction between β2-AR and Nbs

**Figure 2:**
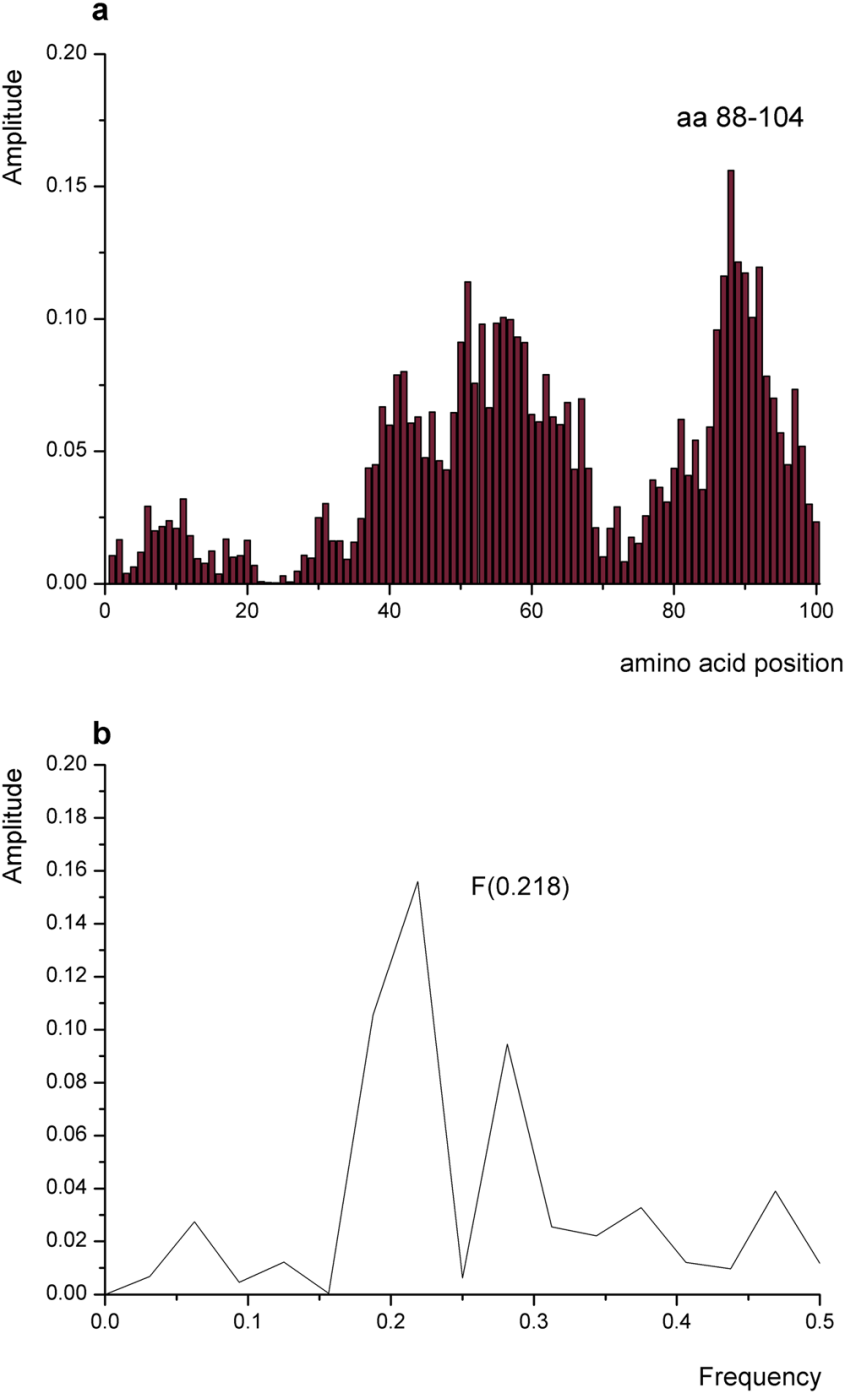
Mapping of the putative interaction sites of β_2_-AR and Nb71. (**a**) Position of the domain in the primary structure of Nb71 (residues 88–104). (**b**) The IS of P3, which is 17 aa long.

Next, a computational peptide scanning analysis was performed to define linear protein regions of the β_2_-AR that exhibited the greatest contributions to the amplitude and signal-to-noise ratio at the characteristic frequency and therefore were responsible for the interaction(s) described by the particular spectral characteristic. By performing computational peptide scanning of Nb71 at F(0.216), we identified the region encompassing amino acid residues (aar) 88–104 (Fig. 2a) as essential for the information represented by this frequency. The identified domain of Nb, denoted by P3, was the key domain responsible for the interaction with the receptor. It was a peptide of 17 aa, EDTAVYYCNANWDLLSD. As shown in Fig. 2b, P3 had an amino acid sequence that reflected common informational properties shared with Nb71. The identified NDP was proposed to be a mimetic of Nb71 and assumed sufficient for the interaction with the agonist-bound β_2_-AR and for stabilizing an active receptor conformation. P3 is located inside CDR3, consistent with some previous findings. First, highly diverse CDR3 regions in all antigen receptors are suggested to be the key determinants of specificity in antigen recognition^27^. Second, an antibody uses only a single loop, its CDR3, to interact directly with the antigen^28^. Because CDR3 within Nb80 is responsible for most of the binding interactions^16^ and because the peptidomimetics of the CDR3 loop were likely sufficient for binding to the receptor and inhibiting the interaction of β_2_-AR with its intracellular GPCR-interacting proteins, such as Gαs and β-arrestins, the peptidomimetics of CDR3 structurally mimicked the CDR3 loop of an Nb^29^.

### Molecular docking of peptides

CABS-dock docking results were the output of the 1,000 top conformations of β_2_-AR-peptide complexes, along with the 10 best and individual trajectories for each final conformation. We chose a complex where the peptide was docked in the intracellular loop using the β_2_-AR-Nb80 binding pattern as a reference. The list of aar and interactions observed in the β_2_-AR-Nb80 complex (PDB 3P0G crystal structure)^16^ are presented in Table 1. These data guided us in selecting properly docked conformations of our peptides. Finally, the best conformations of our four peptides were isolated. Since only one (P3) out of four peptides had reported experimental activity, only P3 data are presented. However, the sole use of flexible ligand docking is not the most reliable method to estimate complex stability and ligand binding affinity; therefore, the obtained complexes were subjected to MD simulations and free energy calculations.

**Table 1:** Interactions between Nb80 and β_2_-AR from the PDB structure 3P0G (A chain represents β_2_-AR, and B chain represents peptide).

Name	Distance	Category	Type	From	From Chemistry	To	To Chemistry	Angle XDA	Angle DAY	Theta	Theta22	Gamma	Closest Atom Distance
A:ARG131:NH2-B:GLU106:OE2	4.55504	Electrostatic	Attractive Charge	A:ARG131:NH2	Positive	B:GLU106:OE2	Negative						
A:ARG131:CD-B:VAL103:O	2.7792	Hydrogen Bond	Carbon Hydrogen Bond	A:ARG131:CD	H-Donor	B:VAL103:O	H-Acceptor	96.039	138.559				
B:SER30:CB-A:ALA226:O	3.76186	Hydrogen Bond	Carbon Hydrogen Bond	B:SER30:CB	H-Donor	A:ALA226:O	H-Acceptor	94.333	136.361				
B:HIS52:CE1-A:ILE135:O	2.95275	Hydrogen Bond	Carbon Hydrogen Bond	B:HIS52:CE1	H-Donor	A:ILE135:O	H-Acceptor	102.029	165.557				
A:ARG328:C,O;SER329:N-B:TYR105	4.42188	Hydrophobic	Amide-Pi Stacked	A:ARG328:C,O;SER329:N	Amide	B:TYR105	Pi-Orbitals			31.609	30.163	12.821	3.962
A:ARG131-B:LEU104	5.45175	Hydrophobic	Alkyl	A:ARG131	Alkyl	B:LEU104	Alkyl						
A:VAL222-B:VAL103	5.4757	Hydrophobic	Alkyl	A:VAL222	Alkyl	B:VAL103	Alkyl						
A:ALA226-B:ILE31	5.16574	Hydrophobic	Alkyl	A:ALA226	Alkyl	B:ILE31	Alkyl						
A:ILE278-B:LEU104	5.27015	Hydrophobic	Alkyl	A:ILE278	Alkyl	B:LEU104	Alkyl						
B:VAL103-A:LEU275	4.88306	Hydrophobic	Alkyl	B:VAL103	Alkyl	A:LEU275	Alkyl						
A:PHE139-B:ALA50	4.74445	Hydrophobic	Pi-Alkyl	A:PHE139	Pi-Orbitals	B:ALA50	Alkyl						
A:TYR326-B:LEU104	5.19168	Hydrophobic	Pi-Alkyl	A:TYR326	Pi-Orbitals	B:LEU104	Alkyl						
B:PHE29-A:ALA226	5.29543	Hydrophobic	Pi-Alkyl	B:PHE29	Pi-Orbitals	A:ALA226	Alkyl						
B:PHE29-A:ALA271	4.40895	Hydrophobic	Pi-Alkyl	B:PHE29	Pi-Orbitals	A:ALA271	Alkyl						
B:HIS52-A:PRO138	4.67849	Hydrophobic	Pi-Alkyl	B:HIS52	Pi-Orbitals	A:PRO138	Alkyl						
B:TYR100-A:ILE135	5.39955	Hydrophobic	Pi-Alkyl	B:TYR100	Pi-Orbitals	A:ILE135	Alkyl						
B:TYR105-A:PRO330	5.27514	Hydrophobic	Pi-Alkyl	B:TYR105	Pi-Orbitals	A:PRO330	Alkyl						

### MD simulations

**Figure 3:**
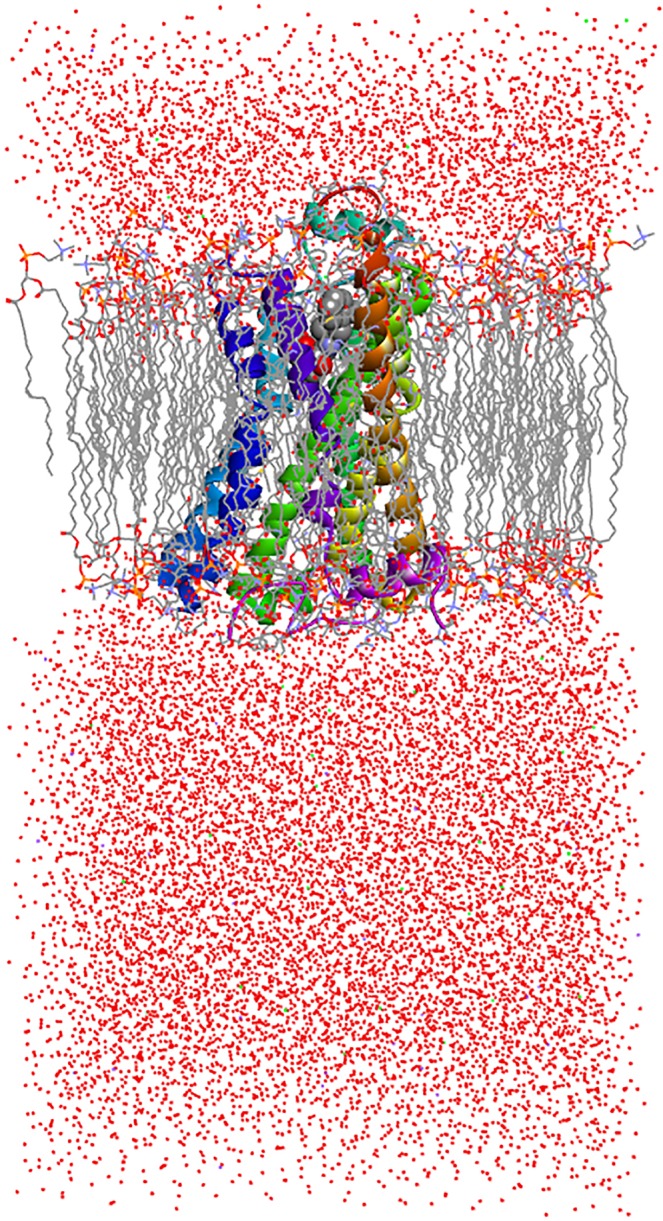
Structure of the β_2_-AR receptor in complex with the cocrystallized agonist P0G (8-[(1R)-2–1-hydroxyethyl]-5-hydroxy-2H-1,4-benzoxazin-3(4H)-one) and the docked peptide P3, prepared for the MD simulation.

**Figure 4:**
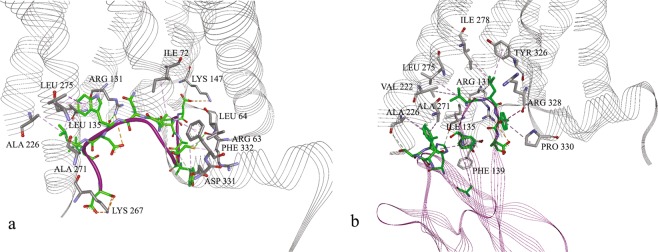
Conformation of (**a**) the docked peptide in β_2_-AR after the first 100 ns production phase and (**b**) Nb80 in β_2_-AR. Marked interactions are the same as observed in the β_2_-AR-Nb80 crystal structure. Orange: electrostatic interactions; purple/grey: hydrophobic interactions. Amino acid residues shown in black belong to the β_2_-AR, while green residues belong to P3.

A prepared complex of β_2_-AR:P3 was subjected to MD simulations to determine its stability. Its structure is shown in Fig. 3. During the 100 ns of the production phase, the β_2_-AR:P3 complex remained stable and intermolecular interactions persisted, with eventual breaking and re-arranging on peptide terminals due to its flexibility. The interactions observed in the β_2_-AR:P3 complex are presented in Fig. 4a and Table 2. The total energy plot and the RMSD plots of both the receptor and peptide showed convergence of the system (Supplementary Figs S1–3). MD alone is neither sufficient to identify the proper conformation of the ligand nor to estimate its affinity for the receptor, and it should be combined with one of the methods used to calculate free energy. The basic idea behind the free energy calculations is to calculate the probability density along the postulated reaction coordinate, and typically, biased sampling is required. Therefore, the next logical step was to select a method that systematically explored the conformational space of the peptide and receptor to calculate their binding free energy values. For that purpose, we chose metadynamics^19,30^, which has also been successfully applied in bimolecular simulations dedicated to the study of protein-protein interactions (reviewed in^20^).

**Table 2:** Interactions between β_2_-AR and P3 after 100 ns of production. A chain: β_2_-AR, B chain: peptide.

Name	Distance	Category	Type	From	From Chemistry	To	To Chemistry	Angle XDA	Angle DAY
A:LYS267:HZ1-B:ASP301:OT1	1.73647	Hydrogen Bond; Electrostatic	Salt Bridge; Attractive Charge	A:LYS267:HZ1	H-Donor; Positive	B:ASP301:OT1	H-Acceptor; Negative	156.026	98.442
A:LYS267:HZ3-B:ASP301:OD1	2.10006	Hydrogen Bond; Electrostatic	Salt Bridge; Attractive Charge	A:LYS267:HZ3	H-Donor; Positive	B:ASP301:OD1	H-Acceptor; Negative	114.335	101.005
A:ARG131:NH1-B:ASP297:OD2	5.09788	Electrostatic	Attractive Charge	A:ARG131:NH1	Positive	B:ASP297:OD2	Negative		
A:LYS147:NZ-B:ASP286:OD1	4.64041	Electrostatic	Attractive Charge	A:LYS147:NZ	Positive	B:ASP286:OD1	Negative		
A:LYS267:NZ-B:ASP301:OT2	4.06921	Electrostatic	Attractive Charge	A:LYS267:NZ	Positive	B:ASP301:OT2	Negative		
A:ARG131:HH12-B:TRP296:O	1.76198	Hydrogen Bond	Conventional Hydrogen Bond	A:ARG131:HH12	H-Donor	B:TRP296:O	H-Acceptor	165.137	162.749
A:LYS147:HN-B:ASP286:OD2	1.75273	Hydrogen Bond	Conventional Hydrogen Bond	A:LYS147:HN	H-Donor	B:ASP286:OD2	H-Acceptor	167.533	133.069
A:SER329:HG1-B:CYS292:O	1.73294	Hydrogen Bond	Conventional Hydrogen Bond	A:SER329:HG1	H-Donor	B:CYS292:O	H-Acceptor	168.889	131.481
B:TYR290:HH-A:ASP331:OD2	1.62197	Hydrogen Bond	Conventional Hydrogen Bond	B:TYR290:HH	H-Donor	A:ASP331:OD2	H-Acceptor	167.973	111.859
A:ARG131:HD1-B:ASN295:OD1	2.38653	Hydrogen Bond	Carbon Hydrogen Bond	A:ARG131:HD1	H-Donor	B:ASN295:OD1	H-Acceptor	159.279	95.278
A:ARG131:HD2-B:TRP296:O	2.61421	Hydrogen Bond	Carbon Hydrogen Bond	A:ARG131:HD2	H-Donor	B:TRP296:O	H-Acceptor	120.492	142.836
A:THR146:HA-B:ASP286:OD2	2.46319	Hydrogen Bond	Carbon Hydrogen Bond	A:THR146:HA	H-Donor	B:ASP286:OD2	H-Acceptor	135.275	147.36
A:SER329:HB1-B:ASN293:O	2.75817	Hydrogen Bond	Carbon Hydrogen Bond	A:SER329:HB1	H-Donor	B:ASN293:O	H-Acceptor	134.877	117.807
A:SER329:HB2-B:CYS292:O	2.99923	Hydrogen Bond	Carbon Hydrogen Bond	A:SER329:HB2	H-Donor	B:CYS292:O	H-Acceptor	91.116	163.907
B:CYS292:HA-A:ASP331:OD2	2.74226	Hydrogen Bond	Carbon Hydrogen Bond	B:CYS292:HA	H-Donor	A:ASP331:OD2	H-Acceptor	160.083	113.094
A:ARG63-B:CYS292	4.49915	Hydrophobic	Alkyl	A:ARG63	Alkyl	B:CYS292	Alkyl		
A:ILE135-B:LEU298	5.36325	Hydrophobic	Alkyl	A:ILE135	Alkyl	B:LEU298	Alkyl		
A:PRO138-B:LEU299	4.86055	Hydrophobic	Alkyl	A:PRO138	Alkyl	B:LEU299	Alkyl		
A:ALA226-B:LEU298	4.37024	Hydrophobic	Alkyl	A:ALA226	Alkyl	B:LEU298	Alkyl		
A:ALA271-B:LEU298	5.18113	Hydrophobic	Alkyl	A:ALA271	Alkyl	B:LEU298	Alkyl		
B:CYS292-A:LEU64	4.90786	Hydrophobic	Alkyl	B:CYS292	Alkyl	A:LEU64	Alkyl		
B:ALA294-A:ILE72	4.72223	Hydrophobic	Alkyl	B:ALA294	Alkyl	A:ILE72	Alkyl		
A:PHE332-B:ALA294	5.11125	Hydrophobic	Pi-Alkyl	A:PHE332	Pi-Orbitals	B:ALA294	Alkyl		
B:TRP296-A:ARG131	4.40471	Hydrophobic	Pi-Alkyl	B:TRP296	Pi-Orbitals	A:ARG131	Alkyl		
B:TRP296-A:LEU275	5.07559	Hydrophobic	Pi-Alkyl	B:TRP296	Pi-Orbitals	A:LEU275	Alkyl		
B:TRP296-A:ARG131	3.87722	Hydrophobic	Pi-Alkyl	B:TRP296	Pi-Orbitals	A:ARG131	Alkyl		
B:TRP296-A:LEU275	4.60971	Hydrophobic	Pi-Alkyl	B:TRP296	Pi-Orbitals	A:LEU275	Alkyl		

### Metadynamics simulations

We performed well-tempered metadynamics simulations of the β_2_-AR:P3 complex after the MD production phase to estimate the binding free energy between β_2_-AR and the peptide. For the centroid of β_2_-AR aar, we chose the backbone carbon atoms Arg63, Asn69, Arg131, Ile135, Tyr141, Thr274, Ser329 and Pro330. We chose all residues of the peptide. The initial distance between the two centroids in the docked conformation was 4,890 Å, and we altered this distance by up to 60 Å. The choice of protocol was not trivial, and the main reason was the flexibility of the peptide ligand. The peptide itself possessed high flexibility, giving rise to slow equilibration and a noisy potential of mean force (PMF). After numerous preliminary metadynamics simulation experiments, we concluded that extraction of the peptide to an intracellular water layer along the z-axis of the system, parallel to the axis of the receptor and perpendicular to the cell membrane was an appropriate protocol. A good starting peptide conformation was identified by assessing its conformation after 100 ns of production where the P3 preserved its orientation and a similar binding pattern that between β_2_-AR and Nb80. (Fig. 4a,b, Tables 1 and 2). For further confirmation of stability after the production phase, we plotted the RMSD values of receptor and peptide (Supplementary Figs S2 and 3). Regarding the stability of β_2_-AR during the production and metadynamics phases, we plotted the distance between the backbone carbon atoms of Trp68 (TM3) and Ala271 (TM6) during production and the metadynamics phase (Supplementary Figs S4 and 5), the RMSDs of the receptor and the total energy profile (Supplementary Figs S6 and 7). The changes in distance during both phases showed the stability of the active conformation, and the values were within the limits of the reference values: 10.766 Å for the inactive conformation and 18.084 Å for the active conformation of β_2_-AR (values from PDB structures 2RH1 and 3P0G, respectively).

**Figure 5:**
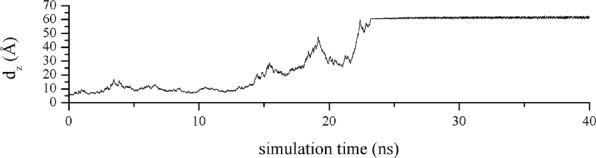
β_2_-AR:P3 distance evolution during metadynamics simulation.

**Figure 6:**
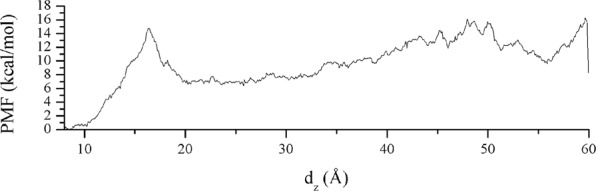
Evolution of potential of mean force during the metadynamics simulation of the initially docked peptide conformation.

The distance evolution, *i.e*., its projection on the z-axis between receptor and peptide, is presented in Fig. 5. Regarding the PMF energy profile, the PMF energy initially increased to ~15 kcal/mol until the distance reached approximately 17 Å in the initial bound conformation as shown in Fig. 6. This energy change originated from the breaking of non-bonded interactions between aar of the receptor and peptide and peptide stretching due to the applied force on the peptide and its flexibility. During metadynamics, receptor-peptide interactions are gradually broken and re-formed. Peptide conformational changes increases the conformational energy, resulting in a very noisy PMF output. After the distance exceeds 17 Å, the PMF slowly decreases because of complete transfer of the peptide to the unbound form and convergence of the internal conformational energy of the peptide, forming a stable PMF area between 20 and 30 Å. After pushing the peptide to the upper boundary, the PMF energy continues to rise as a result of continuous peptide stretching. Afterwards, the peptide is shifted to the upper cell boundary and remains there (Fig. 5). The PMF profile is presented in Fig. 6. The binding free energy value is the negative difference between the initial and final states of PMF evolution. We averaged the stable PMF area between 20 and 30 Å to calculate the binding free energy while avoiding metadynamics artefacts and eliminating noise in the energy. The corresponding PMF difference, according to descriptive statistical analysis, was 7.23 kcal/mol; *i.e*., we estimated the binding free energy of the β_2_-AR:P3 complex as ΔG = −7.23 kcal/mol, or Ki = 7.9 μM at 310 K. The standard error of the calculation was 0.04 kcal/mol, so the final result was written as ΔG = (−7.23 ± 0.04) kcal/mol, or Kd = (7.9 ± 0.5) μM.

The peptide conformational energy minima were meant to be achieved during the 100 ns simulation time. However, due to high conformational freedom of the peptide producing a noisy PMF profile, we had no choice but to select an area within approximately 20 to 30 Å to average the PMF, as it represents the unbound peptide form. The complete metadynamics simulation movie is in Supplementary Material S8, and the non-peptide-bound form is observed at approximately 00:00:07. We are aware that convergence of PMF cannot be reached using only one starting peptide conformation and only one binding/unbinding event, but the good agreement with experimental results (MST and BRET) and limited access to GPU resources prompted us to keep this approach and consider the obtained result as acceptable. In this respect, we considered the calculated potential of mean force as semiquantitative. We feel that for fully converged potential of mean force one would require a simulation length of several tens of microseconds, which exceeds available computer power.

In subsequent steps, we attempted to provide experimental evidence for the computationally characterized interaction between the β_2_-AR and P3.

### Circular dichroism (CD)

**Figure 7:**
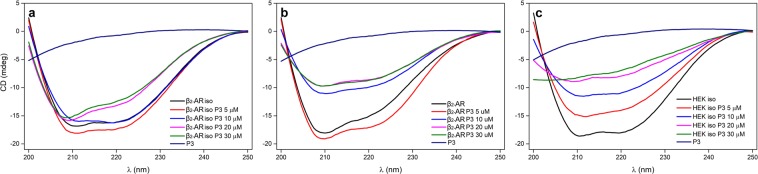
CD spectra of cell lysates prepared from β_2_-AR-transfected/untransfected HEK-293 cells incubated with different concentrations of P3 in the presence or absence of the agonist isoproterenol. Lysates of HEK-293 cells transiently transfected with β_2_-AR/Rluc8 (a and b) and mock-transfected (control) HEK-293 cells (**c**) were titrated with P3 in the presence (**a**,**c**) or absence of the β_2_-AR agonist isoproterenol (**b**). In the presence of agonist, the cell lysate was preincubated with 1 µM isoproterenol, and then P3 was added at a final concentration of 5, 10, 20 or 30 µM. The CD spectrum of P3 (P3, dark blue line) in buffer is shown for comparison. All experiments were performed in PBS, pH 7.2, containing 0.1% DMSO at 25 °C.

Based on the CD spectra for P3 recorded in the far-UV range, this peptide does not have a distinctive secondary structure and is rather unstructured (Fig. 7, dark blue line). Experimental evidence from CD spectropolarimetry for the computationally characterized interaction between β_2_-AR and P3 is presented in Fig. 7. Cell lysates from untransfected (control) HEK-293 cells and HEK-293 cells transfected with β_2_-AR/Rluc8 were titrated with P3 in the presence or absence of the β_2_-AR agonist isoproterenol. Comparison of the graphs in Fig. 7 reveals that P3 strongly interacted with cell lysates prepared from β_2_-AR/Rluc8-transfected HEK-293 cells that were preincubated with the β_2_-AR agonist isoproterenol (Fig. 7a) relative to its interaction with lysates from either unstimulated β_2_-AR transfected HEK-293 cells (Fig. 7b) or isoproterenol-stimulated, untransfected HEK cells (Fig. 7c). This claim is based on the hypothesis that all P3 is bound to β_2_-AR and then the complex contributes to the entire CD signal. In the case of HEK-293 cells, a high contribution of free P3 to the CD signal was shown at P3 concentrations ≥20 µM (Fig. 7c), particularly at wavelengths below 214 nm. We also concluded that the induced CD signal was higher when β_2_-AR-transfected HEK-293 cells were pretreated with agonist than in unstimulated cells (*cf*. panels a and b in Fig. 7). The only difference between the untransfected and β_2_-AR-transfected HEK-293 cells was that the level of β_2_-AR was significantly higher in transfected HEK-293 cells (confirmed by total luminescence measurements) than in untransfected HEK-293 cells, in which low endogenous expression of the β_2_-AR was indicated by the very sensitive cAMP ALPHAscreen^TM^ assay^31^ and the low expression of the β_2_-AR mRNA^32^.

### Microscale thermophoresis (MST) data

**Figure 8:**
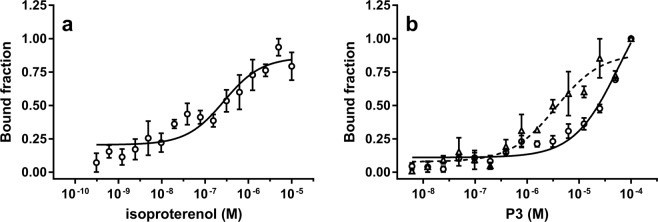
Detection of the interaction of P3 with the β_2_-AR using MST. In the MST experiment, the concentration of β_2_-AR/GFP^2^ was maintained at a constant value by adding equal amounts of cell lysate while increasing the concentration of the known β_2_-AR agonist isoproterenol (control, panel a) or tested NDP, *i.e*., P3, in the absence (panel b, solid line) or presence of 10 µM isoproterenol (panel b, dotted line). After a short incubation, the samples were centrifuged and loaded into MST NT.115 premium capillaries, and the MST analysis was performed using Monolith NT.115pico, as described in the Materials and Methods. The change in the normalized fluorescence (ΔF_norm_) value of each point was divided by the amplitude of the fitted curve to calculate the bound fraction, resulting in values ranging from 0 to 1. Data (mean ± S.E.) were obtained from 3–5 independent measurements.

Next, we used the recently developed MST method to quantify β_2_-AR:P3 interactions. Measurements were performed directly in cell lysates prepared from HEK-293 cells transiently transfected with β_2_-AR tagged with the green fluorescent protein 2 (β_2_-AR/GFP^2^), without the need for a prior protein purification/labelling step. MST-derived dose-response curves for the interactions of isoproterenol and P3 with β_2_-AR are shown in Fig. 8. The EC_50_ of the well-validated β-adrenoceptor agonist isoproterenol for β_2_-AR was in the submicromolar range, *i.e*., 0.275 ± 0.065 µM, which corresponded well to the relatively low affinity of β_2_-AR agonists^10^. On the other hand, P3 displayed EC_50_ values of 58.22 ± 13.59 µM and 3.57 ± 0.81 µM for non-activated and agonist-activated β_2_-AR, respectively. Isoproterenol-activated β_2_-AR thus displayed an approximately 10-fold higher affinity for P3 than unstimulated β_2_-AR (Fig. 8b), corroborating the calculated Ki (7.9 μM) obtained from the metadynamics simulation. This finding is also consistent with the CD spectropolarimetry observations and provides additional experimental evidence for the preference of P3 for agonist-activated β_2_-AR. Similarly, Nb80 efficiently binds to only agonist-occupied β_2_-AR^10^.

### BRET^2^-based β-arrestin 2 recruitment assay

**Figure 9:**
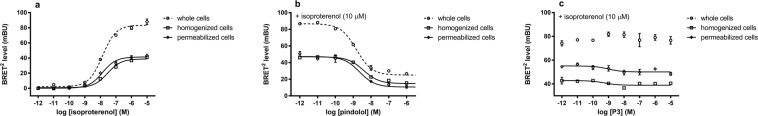
Comparison of ligand-induced BRET^2^ signals. HEK-293 cells were transiently transfected with β_2_-AR/RLuc8 together with the GFP^2^/β-arr2 Arg393Glu, Arg395Glu at a 1:10 cDNA ratio, and the BRET^2^ assay was performed with whole, homogenized or Triton X-100-permeabilized cells. Increasing concentrations of (**a**) the agonist isoproterenol (10^−12^ to 10^−5^ M final concentration), (**b**) the antagonist pindolol (10^−12^ to 10^−6^ M final concentration) and isoproterenol (10 µM final concentration) or (**c**) P3 (10^−12^ to 10^−5^ M final concentration) and isoproterenol (10 µM final concentration) were added to cells, and BRET^2^ was measured as described in Materials and Methods. Data are presented as the means ± S.E. of triplicate measurements.

Because the protein**-**peptide binding energies of the β_2_-AR**:**P3 complex and MST predicted a micromolar-range interaction of P3 with the agonist-activated β_2_-AR, we next evaluated the ability of P3 to interfere with the agonist-induced interaction of β_2_-AR with β**-**arrestin 2, as P3 and β**-**arrestin 2 should compete for the same binding site, and Nb71, which was used as the model to derive P3, is a powerful inhibitor of β-arrestin recruitment to β_2_-AR^14^. Due to the hydrophilic nature of P3 and its presumptive binding to the intracellular receptor site, we modified the previously developed BRET-based β-arrestin 2 recruitment assay^26^ to enable P3 to bind to β_2_-AR. Therefore, we used either homogenized or Triton-X100-permeabilized cells (Fig. 9) instead of whole cells. First, previously characterized β_2_-AR ligands were tested to validate our modified BRET^2^ assay. The maximal agonist-induced BRET signal (BRET_max_) was reduced by approximately 50% when the experiment was performed with homogenized or permeabilized cells relative to the maximal signal when the experiment was performed with whole cells (BRET_max_ of ≈40 *vs*. 80 mBU), whereas the potency of isoproterenol and pindolol remained comparable among the various conditions (see panels a and b in Fig. 9). The obtained BRET^2^ EC_50_ values for the isoproterenol-induced interaction of the β-arrestin 2 Arg393Glu, Arg395Glu mutant with β_2_-AR (13.65 ± 1.98, 28.89 ± 1.98 and 13.74 ± 1.37 nM in whole, homogenized and permeabilized cells, respectively) were also highly consistent with previously reported data^26^. Figure 9b shows the BRET^2^ antagonist dose-response curves generated in the presence of increasing concentrations of the adrenergic receptor antagonist pindolol. The potency of pindolol (IC_50_ of 1.49 ± 0.07, 3.76 ± 0.08 and 2.24 ± 0.22 nM in whole, homogenized and permeabilized cells, respectively) at inhibiting the isoproterenol-induced BRET^2^ signal was consistent with a previously reported range^26^. The hydrophilic NDP P3 showed no effect on the isoproterenol-induced BRET^2^ signal in whole cells, but when either homogenized or permeabilized cells were used, the peptide caused a small dose-dependent reduction (up to 10%) in the isoproterenol-induced BRET^2^ signal (Fig. 9c), with an estimated EC_50_ in a low-nanomolar range, *i.e*., ~1 nM. We presumed that the inability of P3 to effectively compete with β-arrestin 2 for the same binding site was due to its reduced size (less than 25% of the length of the original Nb). Researchers have also postulated that Nb71 inhibits agonist-mediated β-arrestin recruitment to the β_2_-AR by blocking receptor phosphorylation^14^. Therefore, we also hypothesized that the computationally derived NDP of the Nb71 CDR3 is not able to block receptor phosphorylation. The β-arrestin 2 Arg393Glu, Arg395Glu mutant used in our study should bind to the receptor regardless of its phosphorylation status due to the reversed charge of an amino acid in the polar core (Arg393Glu)^33,34^, and this mutation prolongs the lifetime of the receptor:β-arr2 complex due to disruption of β-arrestin 2:AP-2 binding (Arg395Glu^34^). Similarly, peptidomimetics of the Nb80 CDR3 loop were recently shown to only moderately inhibit isoproterenol-induced cAMP production^29^, suggesting that additional residues outside the CDR3 loop are important for effective interference with the Gα/β-arrestin interactions. Alternatively, the relative inefficiency of P3 in the BRET recruitment assay might have been due to the lower affinity of P3 (micromolar range) than purified β-arrestin for phosphorylated β_2_-AR, which is in the nanomolar range (a reported Kd of 1.8 nM)^35^.

In summary, this study presents evidence obtained from a combination of computer-based methodological approaches supported by *in vitro* experimental data used to design and characterize NDPs. It is a small step towards obtaining a better understanding of GPCR dynamics at the molecular level in the context of GPCR interactions with their protein partners. The combined use of experimental and computational techniques represents a powerful framework for achieving progress in this direction and could lead to further modification and optimization of NDPs as efficient modulators of GPCRs and other applications, including drug discovery and therapy.

## Methods

### Materials

Molecular biology and cell culture reagents for were from Sigma-Aldrich (St. Louis, MO, USA) and Gibco Invitrogen Corporation (Breda, The Netherlands). Pindolol and isoproterenol were from Sigma-Aldrich. Coelenterazine 400a from Biotrend Chemikalien GmbH (Köln, Germany). Selected NDPs (P1-P4) were custom-synthesized at Biomatik Corporation, Cambridge, Ontario, Canada.

### Informational spectrum method (ISM)

The principle of the ISM has been thoroughly explained^18,36^ and has been successfully applied to the structure-function analysis of different proteins^18^, the prediction of new protein interactors^37^ and the identification of protein domains responsible for long-range interactions^38^.

### Computational peptide scanning

Computational peptide scanning was utilized to define linear protein regions responsible for the interaction(s) described by the particular spectral characteristic. The sequence of Nb71 was scanned by the ISM algorithm with overlapping windows of different lengths to identify regions with the highest amplitudes at the predefined Fourier frequency.

### Datasets

The sequence of human β_2_-AR used for the bioinformatics analysis was retrieved from the UniProt database with accession number P07550. The sequences of Nbs are presented in US patent US20130137856 and the PDB entry 3P0G FASTA sequence.

### Receptor preparation

The active state crystal structure of the β_2_-AR was obtained from the RSCB protein databank (PDB entry code 3P0G). All lipids, water molecules, and ions and Nb80 were removed. Only the P0G ligand was retained.

### Molecular docking of peptides

Peptide-protein docking was conducted using the online CABS-dock server^39^. Using the given protein receptor 3D structure, binding site and peptide sequence, a docking search for the binding site is performed that allows for full flexibility of the peptide and small fluctuations in the receptor backbone. The output of the simulation is the three-dimensional coordinates of the protein in complex with the ligand accompanied by full docking process trajectories and CABS force field docking scores, including energies of the receptor, ligand and their interaction. The binding site is indirectly defined by unlikely-to-bind regions of β_2_-AR, which exclude all aar regions except intracellular loops. The number of simulation cycles was set to 50. The best solutions, including the peptide in the intracellular space and lowest CABS-dock energy, were selected for further MD simulations.

### Ligand parameterization

Ligand was assigned CGenFF force field atomic charges (ParamChem)^40^ and van der Waals parameters, whereas force constants were obtained from the Hessian equation calculated after geometric optimization of the HF/6–31 G(d) level of theory in Gaussian 03 W^41^. All parameters were generated in the VMD Parameterize extension^42^.

### MD simulations

The β_2_-AR-agonist-NDP complex, with peptide coordinates obtained from the docking output, was inserted into a 70 × 70 Å 2-oleoyl-1-palmitoyl-sn-glyecro-3-phosphocholine (POPC) lipid bilayer. A 10 Å water layer was added from the positive side of the z-axis and 60 Å from the negative side. Bad-contact water molecules were removed from the lipid membrane bilayer using the appropriate tcl script. Additionally, the system was neutralized with 0.15 M NaCl, resulting in a 61,183 (~60,000) atom ensemble. The system was subject to a 10,000 step energy minimization, 250 NVE ps equilibration, and 100 ns NPT MD production. Pressure and temperature were set to 1 bar and 310 K, respectively, using a Berendsen thermostat, and the applied integration step was 1 fs. In all simulations, periodic boundary conditions with particle-mesh Ewald calculations were implemented. The cut-off was set to 12 Å. A CHARMM22^22,23^ force field was used for protein and lipids, and CGenFF^43,44^ was used for ligands.

### Metadynamics simulation

Metadynamics is a powerful method for calculating free energy. It was initially developed by Laio and Parrinello^19^ and later improved to well-tempered metadynamics^20^. Before metadynamics simulation, one must determine the collective variables that will be varied during simulation and in regard to which the PMF will be calculated. We chose one variable, the distance between centroids of protein amino acid (aa) and peptide residues. Their atoms belonged to backbone C of the binding aar in the intracellular loops of the receptor and all residues in the peptide. The lower boundary (minimal distance value) was set to the initial distance between centroids of receptor-ligand atom groups, which was obtained from the coordinates of the docked structure optimized by MD production. The upper boundary (maximal value) was set to the distance at which the peptide was located sufficiently far away from the receptor in the water layer close to the side of the PBC cell. In this way, we ensured that no intermolecular interactions between the protein and peptide were present along all three coordinate axes. During our metadynamics simulation, the peptide pushed towards the intracellular water layer, parallel to the z-axis of the cell and perpendicular to the cell membrane. The resulting change in free energy between initial and equilibrated states of the peptide in the water layer was designated the binding free energy of the complex. Collective variable trajectory frequency (frequency of generating free energy files) was set to 10,000 ps. The lower wall constant (lowest value of applied force, units in kcal/(mol*A)) was set to 120.0, the upper wall constant (highest value of applied force) was set to 180.0, and width (the force resolution) was set to 0.1. For the main atoms, we selected backbone carbon atoms from the receptor, and for the reference, we selected backbone carbon atoms from the peptide. The hill weight (amount of PMF energy that is gradually added to a system during simulation) was set to 0.1 kcal/mol, the hill width was set to 1.0 Å, and the new hill frequency was set to 100 ps. Bias temperature was set to 1550 K. All free energy files generated during simulation were collected. The total metadynamics simulation time was 40 ns, and the integration step was 1 fs. Descriptive statistical analysis of PMF and the corresponding Figures was performed using Origin 8 (OriginLab Corporation, Northampton, MA, USA).

### Fusion constructs

Human HA-tagged β_2_-AR (HAβ_2_-AR) cDNA and human β-arrestin 2 N-terminally tagged with the green fluorescent protein variant 2 GFP^2^ (GFP^2^/β-arr2) were from cDNA Resource Center (www.cdna.org) and from BioSignal Packard Inc., Montreal, Canada, respectively. C-terminal *Renilla luciferase 8* (RLuc8) tagged HAβ_2_-AR (β_2_-AR/RLuc8), C-terminal GFP^2^-tagged β_2_-AR (β_2_-AR/GFP^2^) and mutant GFP^2^/β-arr2 (GFP^2^/β-arr2 Arg393Glu, Arg395Glu) were described in previous studies^26,45,46^.

### Cell culture and transfection

HEK-293 cells (European Collection of Animal Cell Cultures, Salisbury, UK) were routinely maintained and passaged as described previously^45,47^. Transient transfections were performed when cells reached ~90% confluence using the Lipofectamine^®^-*Plus*™ Reagent. The expression levels of RLuc8- and GFP^2^-tagged constructs were monitored by recording total luminescence and fluorescence as previously described^48^.

### Circular dichroism (CD)

All CD spectra were recorded on a Jasco J-1500 spectropolarimeter at 25 °C using a 1 mm quartz cuvette in the UV range of 250–200 nm. The CD spectra were measured every 0.5 nm with a scanning rate of 10 nm/min. Lysed cells (A_280_ = 1.5) (HEK-293, β_2_-AR/Rluc8 transfected HEK-293 cells) were dialysed against PBS buffer, pH 7.2, containing 1% glucose and 0.1% DMSO. P3 was solubilized in 0.1% DMSO in DPBS supplemented with Ca^2+^/Mg^2+^, 1 g/L glucose, and 36 mg/L sodium pyruvate buffer. The concentration of the P3 stock solution was 1 mM. CD spectra of a 10-fold dilution of the initial cell lysate in the presence of P3 at final concentrations of 0, 5, 10, 20, and 30 µM were recorded. The density of HEK-293 and β_2_-AR/Rluc8-transfected HEK-293 cells were equal, as determined spectrophotometrically. In the case of agonist addition (10 µM), the cell lysate was incubated with agonist for 5 minutes at 25 °C before the CD spectra were recorded. Each CD spectrum was subtracted from the CD spectrum of the buffer.

### Microscale thermophoresis (MST)

HEK-293 cells plated in a 75 cm^2^ flask were transiently co-transfected with constructs encoding β_2_-AR/GFP^2^ (5.0 μg), and cell lysates were prepared after 48 h as described below. The 75 cm^2^ flasks were transferred to ice, and cells were washed once with ice-cold DPBS. Then, 750 µL of NP40 lysis buffer was added to each 75 cm^2^ flask and incubated on ice for 5 minutes. Cells were scraped from the 75 cm^2^ tissue culture flasks using cell scrapers (Falcon), and detached cells were incubated on ice for an additional 20 minutes. Cell lysates were transferred to cold 1.5 mL tubes and clarified by centrifugation (15,300 rpm for 30 minutes at 4 °C). Next, 700 µL of cleared lysate was transferred to a new cold 1.5 mL tube, and the level of β_2_-AR/GFP^2^ was verified by measuring the total fluorescence. Ten microlitres of cell lysate were used as the target, while the non-fluorescent ligands isoproterenol and P3 were titrated in a series of 1:1 dilutions in MST buffer containing 0.05% Tween 20. Ten microlitres of the serial dilution of the ligand were mixed with 10 μL of the cell lysate. After a short incubation at room temperature, samples were centrifuged and loaded into Monolith™ NT.115 MST Premium Capillaries (NanoTemper Technologies GmbH, Munich, Germany) and measured using a Monolith NT.115pico and MO. The control software was set to room temperature (25 °C) (LED/excitation power setting 95%, MST power setting low). Data were analysed using MO.Affinity Analysis software (version v2.2.4, NanoTemper Technologies) at the standard MST-on time of 5 s and presented as a bound fraction. The change in the normalized fluorescence (ΔF_norm_) value of each point was divided by the amplitude of the fitted curve to calculate the bound fraction, resulting in values ranging from 0 to 1. This approach is independent of both the initial F_norm_ value and the amplitude of the binding curve and thus enabled us to compare the EC_50_ values of interactions with different amplitudes.

### BRET-based β-arrestin 2 recruitment assay

We used a previously described BRET-based βarr2 recruitment assay^26,45,48^, with some modifications. Because P3 binds to intracellular receptor domains, BRET assays were performed using either homogenized cells or permeabilized cells. HEK-293 cells cultured in a 75 cm^2^ flask were transiently co-transfected with constructs encoding β_2_-AR/RLuc8 (0.1 μg) alone or together with the double GFP^2^/β-arr2 Arg393Glu, Arg395Glu mutant (4.9 μg). Homogenized/permeabilized cells were prepared 48 h after transfection. Homogenized cells were washed twice with DPBS, scraped from 75 cm^2^ tissue culture flasks using cell scrapers (Falcon) and pelleted by centrifugation at 1,000 rpm for 5 minutes. The cell pellets were resuspended in 1 mL of DPBS supplemented with Ca^2+^/Mg^2+^, 1 g/L glucose, and 36 mg/L sodium pyruvate, incubated on ice for 10 minutes, and homogenized using a glass homogenizer (BDH).

Cells were permeabilized by adding 0.01% Triton™ X-100 in DPBS and incubating for 15 minutes at 37 °C. Homogenized/permeabilized cells were then diluted in supplemented DPBS such that the luminescence signal in 180 μL of DPBS containing homogenized/permeabilized cells distributed in 96-well microplates (white Optiplate; Packard BioScience, Meriden, CT, USA) was approximately 30,000 arbitrary units. After the coelenterazine 400a was injected (final concentration of 5 μM), the luminescence signals were recorded (TriStar LB 942 microplate reader, Berthold Technologies, Bad Wildbad, Germany). Signals at 410 nm and 515 nm were recorded sequentially, and the 515/410 ratio (BRET^2^ signal) was calculated. The results were reported in milliBRET units (mBU); BRET^2^ ratio × 1000. The expression levels of RLuc8- and GFP^2^-tagged constructs in each experiment were assessed by measuring the total luminescence and fluorescence as described above. Determinations were performed in triplicate. The obtained data were transferred to GraphPad Prism 7.0 (GraphPad software, San Diego, CA, USA) and BRET EC_50_/IC_50_ values (nM ± SEM) generated using a sigmoidal dose-response curve fit.

## Supplementary information

Supplementary material

## Data Availability

All data generated or analysed during this study are included in this published article and its Supplementary Information Files.
