# Supplementary material for: Computational design and characterization of nanobody-derived peptides that stabilize the active conformation of the β2-adrenergic receptor (β2-AR)
Source: Sci Rep. 2019 Nov 12;9:16555. doi: 10.1038/s41598-019-52934-8 (PMC6851183; doi:10.1038/s41598-019-52934-8)
Supplement: Supplementary file 1 — Supplementary material [file 41598_2019_52934_MOESM1_ESM.pdf]

Supplementary material

Computational design and characterization of nanobody-derived peptides that stabilize the active conformation of the β2-adrenergic receptor (β2-AR)

Milan Sencanski[1], Sanja Glisic[1], Marko Šnajder[2], Nevena Veljkovic[1], Nataša Poklar Ulrih[2], Janez Mavri[3], and Milka Vrecl[4*]

[1]Center for Multidisciplinary Research, Institute of Nuclear Sciences VINCA, University of Belgrade, Belgrade, Serbia

[2]Biotechnical Faculty, University of Ljubljana, Ljubljana, Slovenia

[3]Department of Computational Biochemistry and Drug Design, National Institute of Chemistry, Ljubljana, Slovenia

[4]Institute of Preclinical Sciences, Veterinary Faculty, University of Ljubljana, Slovenia

***Corresponding author**

Milka Vrecl, University of Ljubljana, Veterinary Faculty, Institute of Preclinical Sciences, Gerbičeva 60, 1000 Ljubljana, Slovenia

Tel: +386 1 477 9118

Fax: +386 1 283 22 43

E-mail: milka.vrecl@vf.uni-lj.si

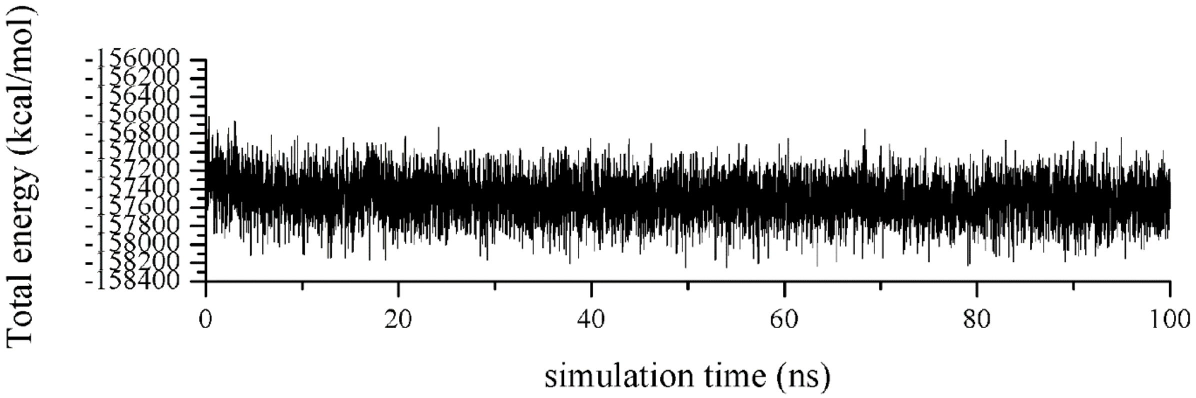

Supplementary Fig. S1. Plot of the total energy *vs.* simulation time during the production phase.

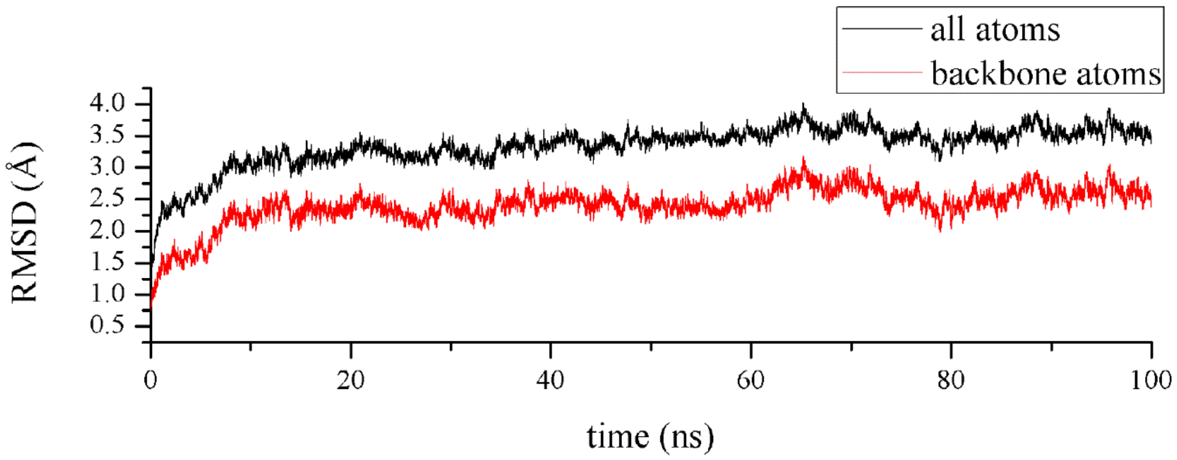

Supplementary Fig. S2. RMSD of receptor atoms during the production phase.

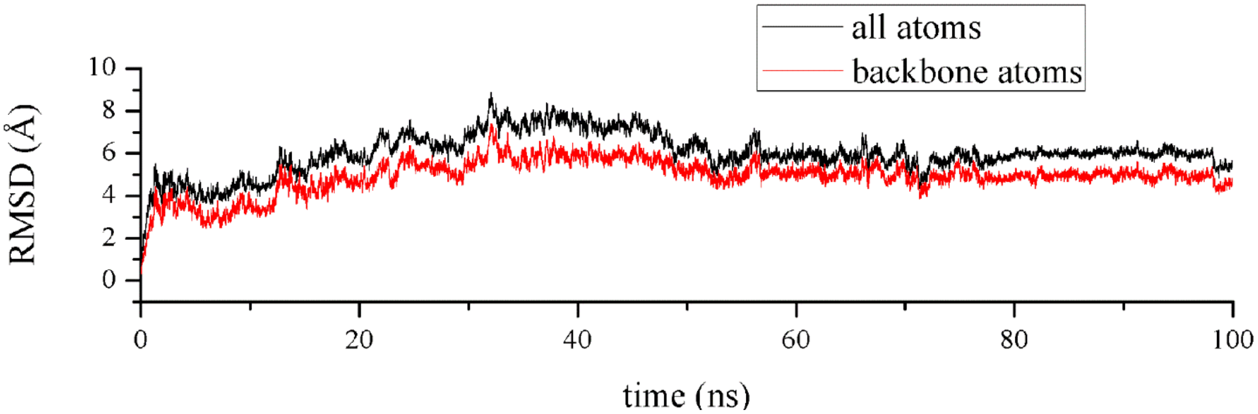

Supplementary Fig. S3. RMSD of peptide atoms during the production phase.

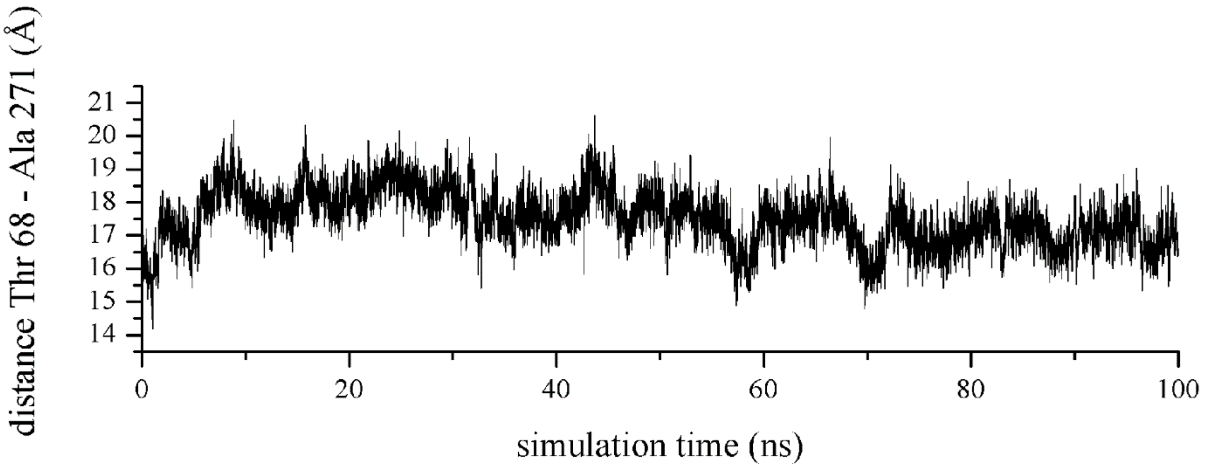

Supplementary Fig. S4. Evolution of the distance between the backbone carbon atoms of β_2-AR Thr68 and Ala271 during the production phase.

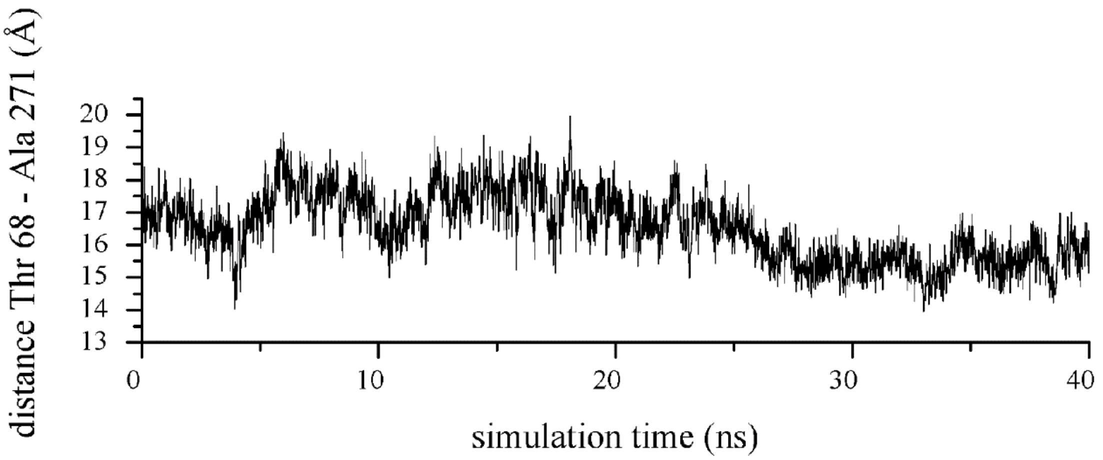

Supplementary Fig. S5. Evolution of the distance between the backbone carbon atoms of Thr68 and Ala271 during the metadynamics phase.

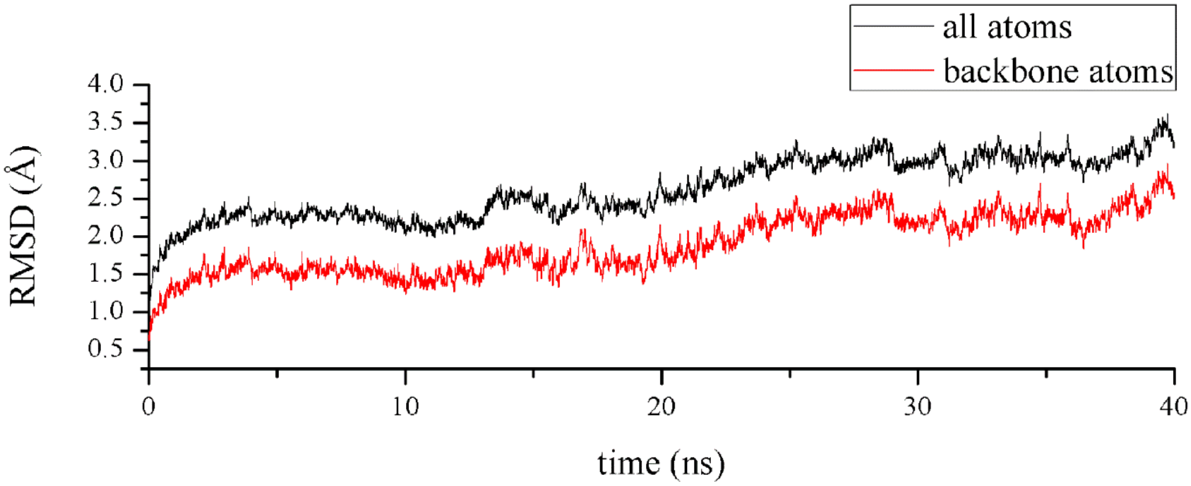

Supplementary Fig. S6. RMSD of receptor atoms during the metadynamics phase.

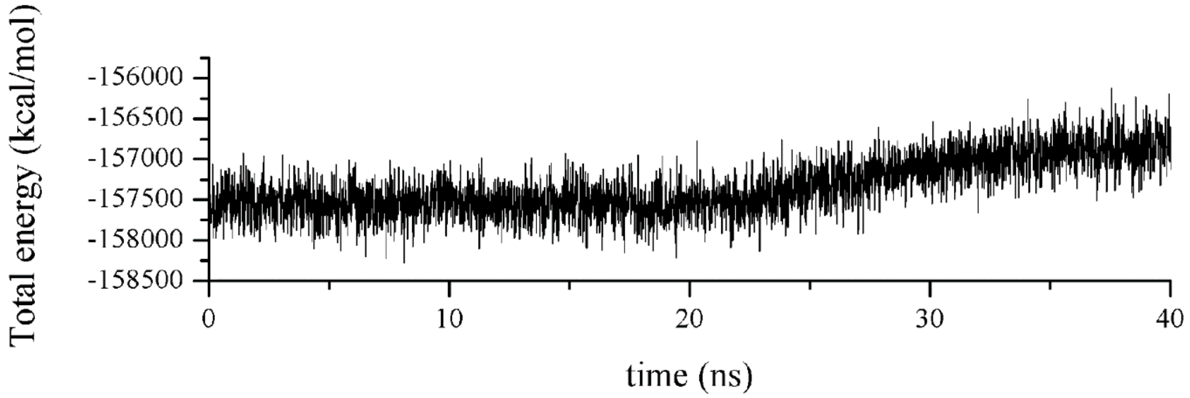

Supplementary Fig. S7. Plot of the total energy *vs.* simulation time during the metadynamics phase.

Supplementary Material S8. Metadynamics simulation movie can be found at:

https://drive.google.com/file/d/1GpTlnJTBN2DOQzRZiB1Xa-dAgYqTzncT/view
